# Supplementary material for: Learning to detect AI texts and learning the limits
Source: PLoS One. 2025 Oct 15;20(10):e0333007. doi: 10.1371/journal.pone.0333007 (PMC12527182; doi:10.1371/journal.pone.0333007)
Supplement: S2 File — All data and scripts used in the study, some additional descriptive statistics, scalable formate figures. (ZIP) [file pone.0333007.s002.tgz › data and scripts/OSF/scripts/analysis/Dependencies/GLS3_difference_vs_Reaction_time_log.pdf]

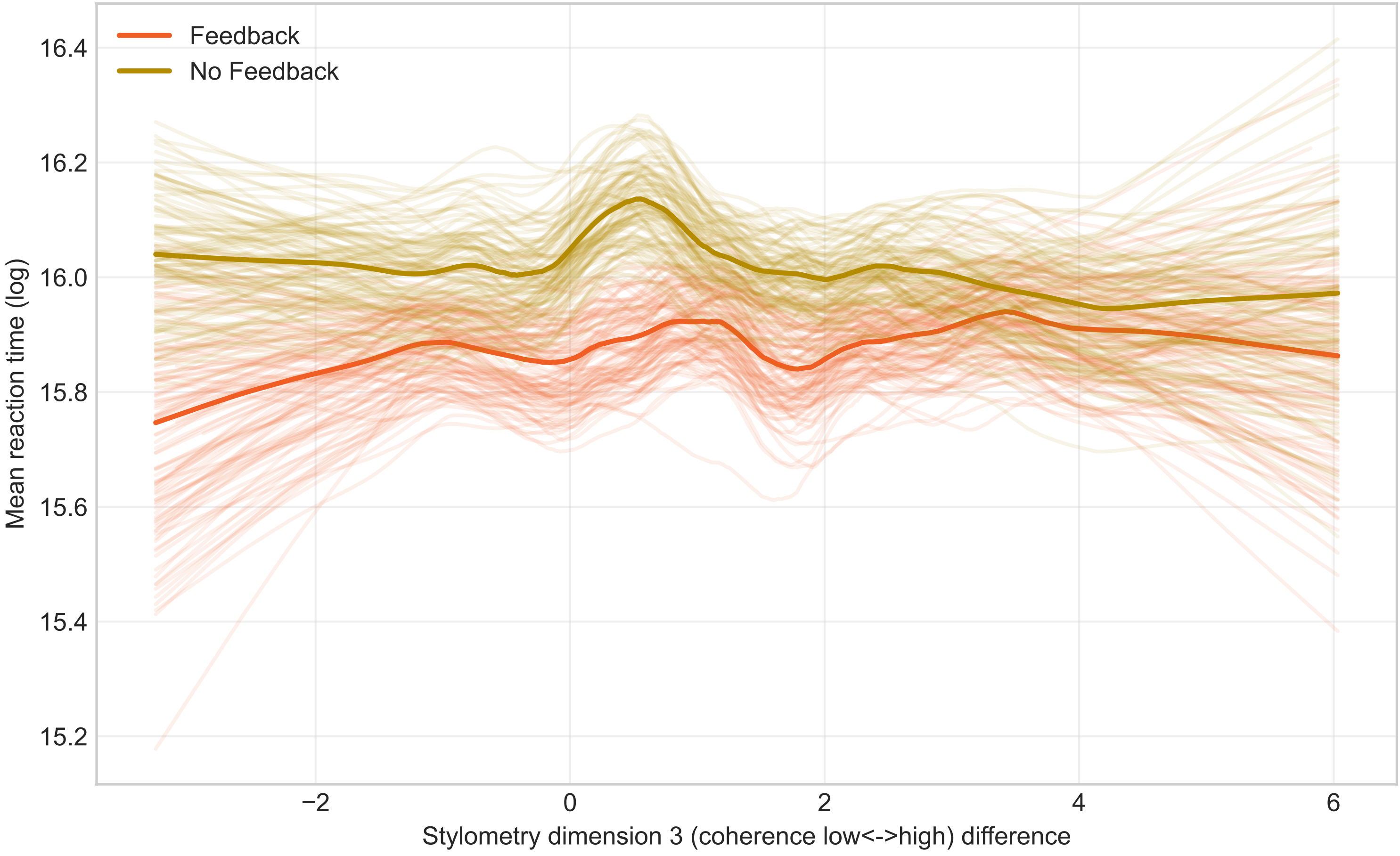
Feedback
No Feedback
16.4
16.2
16.0
15.8
15.6
15.4
15.2
Mean reaction time (log)
−2
0
2
4
6
Stylometry dimension 3 (coherence low<->high) difference
